# Dopamine induces the accumulation of insoluble prion protein and affects autophagic flux

**DOI:** 10.3389/fncel.2015.00012

**Published:** 2015-02-02

**Authors:** Marcio H. M. da Luz, Italo T. Peres, Tiago G. Santos, Vilma R. Martins, Marcelo Y. Icimoto, Kil S. Lee

**Affiliations:** ^1^Department of Biochemistry, Molecular and Cellular Biology, Universidade Federal de São PauloSão Paulo, Brazil; ^2^Biomedicina, Universidade Metodista de São PauloSão Paulo, Brazil; ^3^International Research Center, A C Camargo Cancer CenterSao Paulo, Brazil; ^4^Department of Biophysics, Universidade Federal de São PauloSão Paulo, Brazil

**Keywords:** dopamine, protein aggregation, prion, protein synthesis, autophagy, neurodegeneration

## Abstract

Accumulation of protein aggregates is a histopathological hallmark of several neurodegenerative diseases, but in most cases the aggregation occurs without defined mutations or clinical histories, suggesting that certain endogenous metabolites can promote aggregation of specific proteins. One example that supports this hypothesis is dopamine and its metabolites. Dopamine metabolism generates several oxidative metabolites that induce aggregation of α-synuclein, and represents the main etiology of Parkinson's diseases. Because dopamine and its metabolites are unstable and can be highly reactive, we investigated whether these molecules can also affect other proteins that are prone to aggregate, such as cellular prion protein (PrP^C^). In this study, we showed that dopamine treatment of neuronal cells reduced the number of viable cells and increased the production of reactive oxygen species (ROS) as demonstrated in previous studies. Overall PrP^C^ expression level was not altered by dopamine treatment, but its unglycosylated form was consistently reduced at 100 μM of dopamine. At the same concentration, the level of phosphorylated mTOR and 4EBP1 was also reduced. Moreover, dopamine treatment decreased the solubility of PrP^C^, and increased its accumulation in autophagosomal compartments with concomitant induction of LC3-II and p62/SQSTM1 levels. *In vitro* oxidation of dopamine promoted formation of high-order oligomers of recombinant prion protein. These results suggest that dopamine metabolites alter the conformation of PrP^C^, which in turn is sorted to degradation pathway, causing autophagosome overload and attenuation of protein synthesis. Accumulation of PrP^C^ aggregates is an important feature of prion diseases. Thus, this study brings new insight into the dopamine metabolism as a source of endogenous metabolites capable of altering PrP^C^ solubility and its subcellular localization.

## Introduction

Aberrant protein aggregation is a common hallmark of many neurodegenerative diseases, while a specific protein predominantly aggregates in each type of diseases (Brundin et al., 2010). Mutations that alter amino acid sequence or modifications of side chains by reactive molecules can disrupt the native fold (Tyedmers et al., 2010; Petrov and Zagrovic, 2011). Considering that proteins perform numerous biological activities, the accumulation of their aggregates can be toxic and effective clearance of structurally altered proteins can be essential for cellular survival (Tyedmers et al., 2010).

Most cases of neurodegenerative diseases are idiopathic and protein aggregation can occur without defined mutations or clinical histories that clearly justify the manifestation of the diseases (Alkhuja, 2013; Musiek and Schindler, 2013). This observation raises the possibility that certain endogenous metabolites can induce protein misfolding, and protein aggregates may accumulate upon excessive formation of such metabolites and/or failure of degradation pathways (Morris, 2013). For instance, reactive oxygen species (ROS) are produced as byproducts of several endogenous metabolic pathways, such as mitochondrial respiration and oxidase catalyzed reactions, which include the metabolism of catecholamine (Andreyev et al., 2005; Dikalov, 2011; Kodama et al., 2013). Among catecholamines, dopamine is the one that has the greatest propensity for oxidation (LaVoie et al., 2005). During dopamine metabolism, ROS are generated not only by enzymatic reactions but also by auto-oxidation of dopamine, producing highly reactive dopamine quinone (Asanuma et al., 2003). The accumulation of neuromelanin (which is synthesized from dopamine quinone) throughout the aging process is an indication of the production of dopamine quinone in physiological conditions (Herrero et al., 1993; Kim et al., 2006). Thus, enhanced dopamine metabolism can induce oxidative stress leading to mitocontrial dysfunction and disturbance in protein quality control system (Chen et al., 2008; Hastings, 2009). Although dopamine metabolism has been extensively linked to the aggregation of α-synuclein and Parkinson's disease (Cappai et al., 2005; Lee et al., 2011), dopamine oxidation can also induce the formation of adducts and aggregates of other proteins, such as cytoskeleton proteins and mitochondrial complexes (Van Laar et al., 2009). Moreover, dopamine can also oxidize prion protein *in vitro* in the presence of copper ions (Shiraishi and Nishikimi, 2002; Shiraishi et al., 2005).

Conformational alteration of cellular prion protein (PrP^C^) is an essential event for development of transmissible spongiform encephalopathies (TSE). However, in its normal conformation, PrP^C^ plays important roles in cell growth, differentiation and survival (Linden et al., 2008). Previous studies have demonstrated that PrP^C^ also possesses anti-oxidant properties, but the mechanisms related with this function are still under debate. The expression of PrP^C^ appears to increase during the oxidative stress caused by ischemia and antioxidant defense mechanisms appear to require PrP^C^ expression (McLennan et al., 2004; Beraldo et al., 2013). Direct reactions between PrP^C^ and ROS have also been proposed (Bertuchi et al., 2012), suggesting that PrP^C^ might play roles in scavenging ROS from the extracellular environment. PrP^C^ is highly expressed in neurons and has flexible N-terminal domain enriched by amino acid residues that are more susceptible to oxidation (Linden et al., 2008; Abouelatta et al., 2009). Thus, it is plausible to hypothesize that PrP^C^ can be a preferential target of ROS in the central nervous system with consequent inactivation of ROS activity. PrP^C^ appears to participate in the regulation of dopamine metabolism (Lee et al., 1999; Adjou et al., 2008; Rial et al., 2014), but the role of PrP^C^ in oxidative stress caused by dopamine oxidation has not yet been evaluated. In this study, we investigated the effects of dopamine toxicity on the expression levels, solubility and subcellular localization of PrP^C^, with concomitant analysis of cellular viability, protein synthesis and degradation pathways.

## Materials and methods

### Antibodies and reagents

DMEM Media-Glutamax™-I and fetal bovine serum (FBS) were purchased from Life Technologies. Dopamine hydrochloride, thiazolyl blue tetrazolium bromide (MTT) and 2′, 7′-dichlorofluorescein diacetate (DCFDA) were purchased from Sigma-Aldrich. Complete Protease Inhibitor cocktail tablets were purchased from Roche and Pierce™ phosphatase inhibitor mini tablets were purchased from Thermo Scientific. All primary antibodies were purchased from Cell Signaling, except anti-prion SAF32, which was purchased from Cayman. Horseradish peroxidase conjugated secondary antibodies were purchased from Sigma-Aldrich and Alexa fluorophore conjugated antibodies and CellROX Green reagent were purchased from Life Technologies.

### N2a cell culture

N2a cells were maintained at 37°C in a humidified atmosphere of 5% CO_2_ in DMEM-Glutamax™-I (Dulbecco's Modified Eagle's Medium) supplemented with 10% FBS, Penicillin (100 U/mL) and Streptomycin (100 μg/mL). For dopamine treatment, 4 × 10^4^ cells/cm^2^ were seeded. After 24 h, cells were treated with 50 or 100 μM dopamine diluted in DMEM containing 0.5% of FBS for 24 h.

### Cell viability assay

Cells were treated with dopamine in 96-well plates for 24 h. After the treatment, cells were incubated with 100 μl of 1.2 mM MTT diluted in Krebs solution (NaCl 126 mM, KCl 2.5 mM, NaHCO_3_ 25 mM, NaH_2_PO_4_ 1.2 mM, MgCl_2_ 1.2 mM, CaCl_2_ 2.5 mM, D-glucose 10 mM) for 2 h. The MTT formazan were solubilized in 100 μl of DMSO. Insoluble materials were removed by centrifugation at 20,000 g for 2 min and absorbance of supernatant was measured at 540 nm.

### ROS measurement

Cells were treated with dopamine in 96-well plates for 24 h. After the treatment, cells were incubated with 10 μM of DCFDA or 5 μM CellRox Green in DMEM 0.5% FBS for 30 min. After washing twice with PBS or Krebs solution, the fluorescence was measured using plate reader (Bio-Tek). CellRox Green was also analyzed by Operetta high content screening system (Perkin Elmer).

### Western blot

Cells were lysed with lysis buffer (Tris 100 mM, pH 8.0, NaCl 130 mM, EDTA 10 mM, Triton X-100 1%, sodium deoxycholate 0.5%, complete protease inhibitor cocktail and Pierce phosphatase inhibitor mini tablet), and post-nuclear supernatants (PNS) were collected. The protein content in PNS was quantified using BCA protein assay kit (Thermo Scientific Pierce). Equal amounts of proteins were resolved by SDS-PAGE and transferred to PVDF membrane. For immunodetection, membranes were incubated with TBS-T (Tris 50 mM, NaCl 150 mM, Tween-20 0.1% pH 7.4) containing 5% BSA for 1 h. Then, the membranes were incubated with primary antibody for 1 h at room temperature or for 16 h at 4°C, then washed three times with TBS-T. Secondary antibody conjugated with peroxidase and SuperSignal™ West Pico Chemiluminescent Substrate were used to detect antigens labeled with specific antibodies. Images were digitalized and quantified using Alliance mini 4 m (UVITEC Cambridge). For each protein, at least three independent experiments were performed. In every experiment, the band intensity of the proteins of interest (PI) was normalized by the intensity of the GAPDH bands. Western blot is semi-quantitative assay and direct comparison of PI/GAPDH ratio between independent experiments is not possible. Thus, in order to enable this comparison, the average of PI/GAPDH ratio of three experimental groups (cells treated with 0, 50, and 100 μM of dopamine) was set as 100%, and the percentage of each group was calculated in each independent experiment. The mean percentage of all independent experiments was plotted with respective 95% confidence interval (CI95). An example of PrP^C^ expression analysis was shown in supplementary data.

### Ultracentrifugation

Post-nuclear supernatants were incubated with 1% sarkosyl for 10 min on ice, then ultracentrifuged for 2 h at 100,000 × g and at 4°C. Pellets were dissolved in sample buffer 4x (8% SDS, Tris HCl 250 mM, pH 6.8, 40% Glycerol, 0.08 mg/ml bromophenol blue and 1.4 M β-mercaptoetanol), and supernatants were incubated with 4 volumes of methanol for 2 h at −20°C for protein precipitation. After centrifugation for 20 min at 25,000× g and 4°C, methanol precipitated pellets were dissolved in Sample Buffer 4x. The level of PrP^C^ in each fraction was assessed by western blot. To combine the results of multiple independent experiments, percentage of PrP^C^ observed in each sample was calculated considering the average of six samples (Pellets and supernatants of 0, 50, and 100 μM) as 100%. This normalization was necessary because the absolute value of the band intensity is not comparable from one blot to another. The mean percentage of all independent experiments was plotted with respective CI95.

### Purification of biotinylated cell surface proteins

Cell surface proteins were biotinylated using Pierce Cell Surface Protein Isolation Kit (Thermo Scientific). The cells were harvested at time zero or after 1 h incubation in the presence or absence of 50 μM of dopamine. Biotinylated proteins were purified from the cell lysates using NeutrAvidin-agarose, and PrP^C^ was detected by western blot using SAF32 antibody.

### Immunofluorescence

Cells were fixed with 3.7% paraformaldehyde diluted in PBS (pH 7) for 5 min and then with alkalinized 3.7% paraformaldehyde (pH 10) for 15 min. After washing twice with PBS, excess of paraformaldehyde was inactivated with glycine 100 mM and cells were permeabilized with Triton X-100 0.1% diluted in PBS for 10 min. To avoid unspecific bindings, cells were incubated with 1% BSA diluted in PBS for 1 h and then with primary antibodies diluted in PBS containing Triton X-100 0.1% for 1 h. After washing three times with PBS, cells were incubated with secondary antibody conjugated with Alexa 488 or Alexa 594 fluorophore for 1 h and then washed five times with PBS. Average images of six frames were acquired for each visual field using confocal microscope (*Leica TCS SP8)*. Images were analyzed using Image J 1.48r. In each image, total area stained by anti-PrP^C^ was defined as 100% and percentage of the area co-stained by anti-LC3-II was assessed using Colocalization Threshold plugin with default parameters (Bolte and Cordelieres, 2006). To estimate the distribution of PrP^C^ in intracellular vesicles with distinct sizes, we counted the number of PrP^C^-positive particles and then categorized them by their size. The percentage of each category was calculated in relation to total number of particles counted in each group.

### Oligomerization of recombinant PrP (rPrP)

rPrP was prepared as described elsewhere (Zahn et al., 1997). The oligomerization was performed in 20 μl of Tris buffer (10 mM pH 7.4) containing 10 μg rPrP and dopamine at designated concentrations. This mixture was incubated for 24 h at 37°C protected from the light unless otherwise specified. Sodium metabisulfite (400 μM) was added whenever necessary. The product of oligomerization was separated in 4–20% gradient SDS polyacrylamide gel and revealed by silver staining.

### Statistical analysis

All experiments, except ROS measurement and biotinylation of cell surface proteins, were repeated 3–5 times. For ROS measurement, one experiment was performed in triplicate for each dye and detection methods. Mean of independent experiments or replicates was plotted with respective CI95. The groups were considered different when their CI95 did not overlap. In this criterion, *p-*values are estimated to be lower than 0.01 (Cumming et al., 2007).

## Results

### Dopamine alters the expression level of unglycosylated PrP^C^

**Figure 1:**
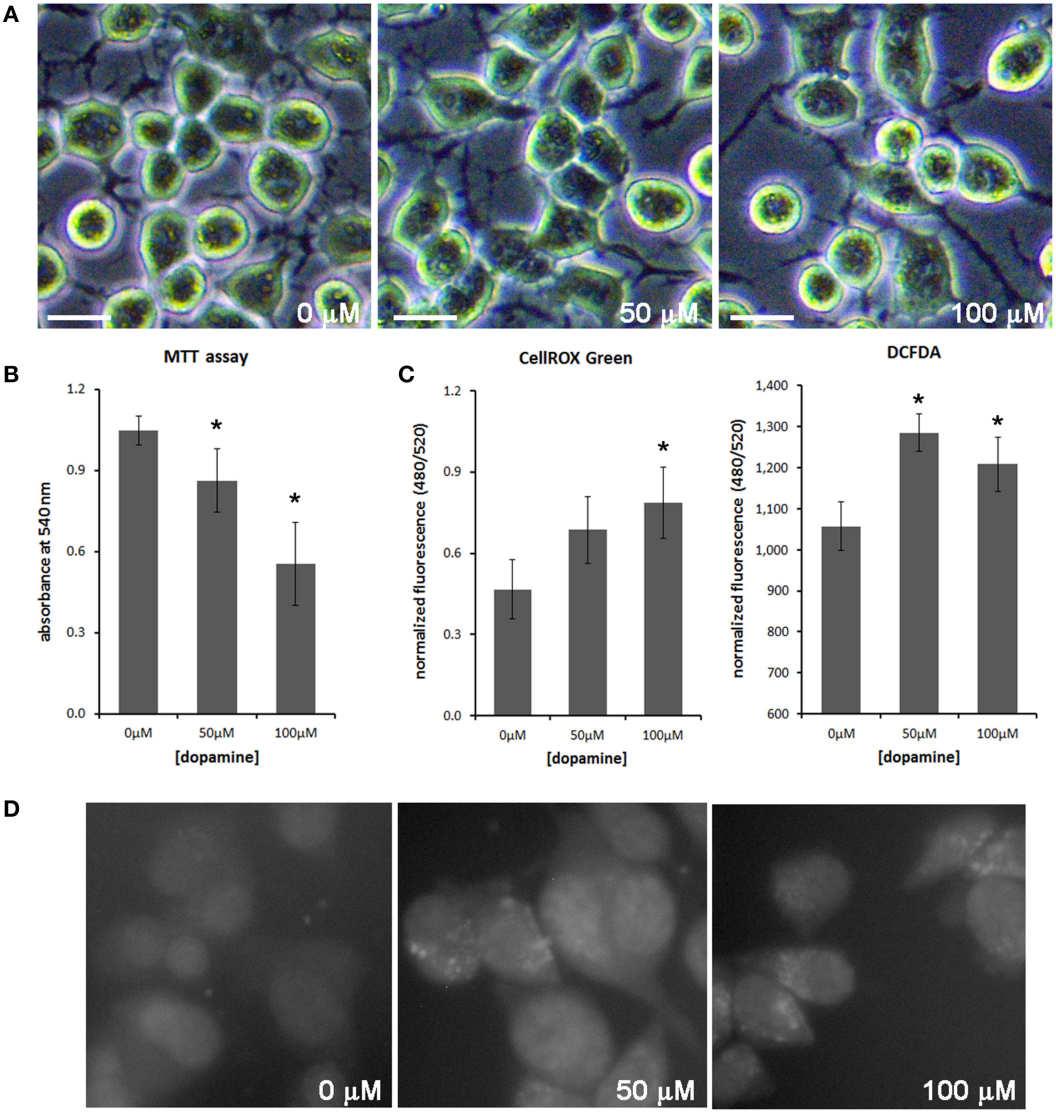
**Dopamine reduced cell viability and increased ROS production**. The morphology of N2a cells was not altered with dopamine treatment for 24 h **(A)**, but the viability assessed by the formation of MTT formazan was reduced after dopamine treatment **(B)**. Mean of four independent experiments was represented with respective CI95 **(B)**. ROS production was evaluated using CellROX Green reagent and DCFDA. Fluorescence was measured by high-throughput plate reader and normalized by total protein contents/well or by cell viability. Mean of triplicate was plotted with respective CI95 **(C)**. CellROX Green was also analyzed by high content cell analyzer. Representative field images are shown **(D)**. ^*^no overlap of CI95 between indicated group and control group (0 μM). scale bar = 20 μM.

Treatment of neuronal cells with high concentration of dopamine for a prolonged period (typically 24 h) is known to evoke cytotoxic effects (Gomez-Santos et al., 2003; Yamakawa et al., 2010). To evaluate the effect of dopamine cytotoxicity on PrP^C^, we treated N2a cells with 50 and 100 μM of dopamine for 24 h. This treatment did not induce morphological alterations, but cell viability was gradually reduced as dopamine concentration was increased (Figures 1A,B). Dopamine treatment also induced ROS production as assessed by CellROX Green reagent and 2′,7′-dichlorofluorescein diacetate (DCFDA), compounds that show enhanced fluorescence upon oxidation. Both reagents showed similar results in high-throughput plate reader (Figure 1C), but the increase of CellRox fluorescence induced by 50 μM of dopamine was not statistically significant. This subtle difference between two dyes can be explained by their different reactivity. CellROX Green was also analyzed by high content cell analyzer. As shown in Figure 1D, brighter fluorescent spots were observed in cells treated with 50 and 100 μM of dopamine. According to the manufacturer, this pattern is due to the primary location of CellROX in the nucleus and mitochondria upon oxidation.

**Figure 2:**
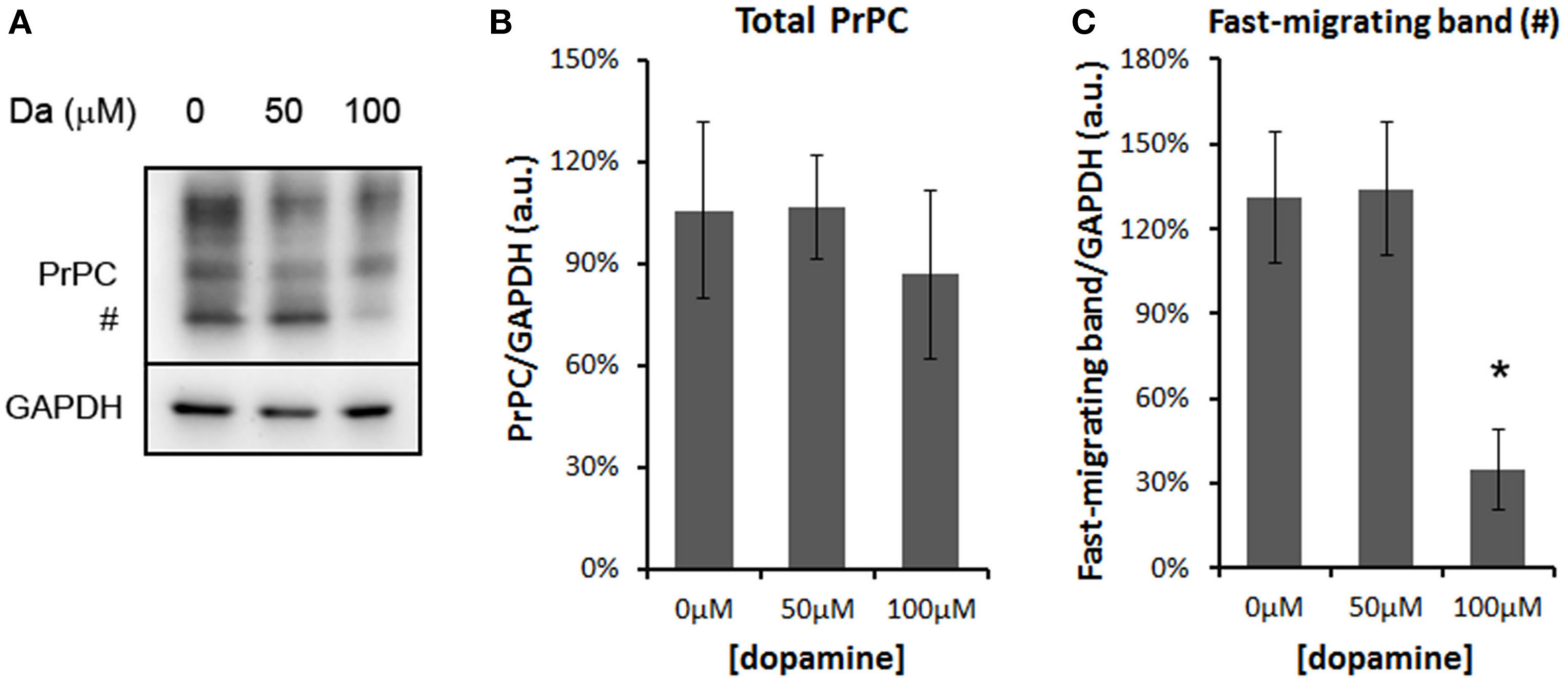
**Dopamine reduced the expression level of unglycosylated PrP^C^**. Treatment of N2a cells with dopamine for 24 h did not alter overall expression of PrP^C^, but reduced the level of fast-migrating unglycosylated band of PrP^C^ (#, **A**). The intensity of total PrP^C^ **(B)** or fast-migrating band **(C)** was normalized by respective GAPDH band. Means of 5 independent experiments were plotted with respective CI95. ^*^no overlap of CI95 between indicated group and control group (0 μM).

**Figure 3:**
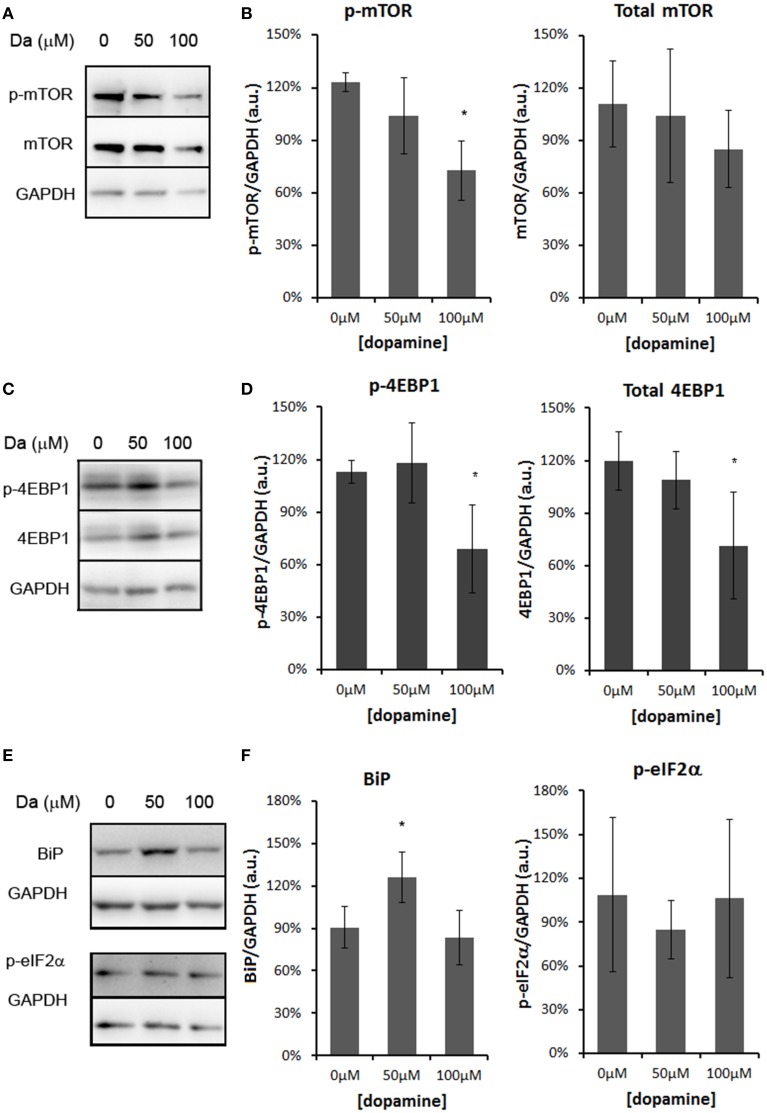
**Dopamine treatment compromised protein synthesis**. The level of phosphorylated mTOR (p-mTOR) was reduced by 100 μM of dopamine, while total mTOR was not altered **(A,B)**. The level of phosphorylated 4EBP-1 (p-4EBP-1) and total 4EBP1 was reduced by 100 μM of dopamine **(C,D)**. The level of BiP was induced by 50 μM of dopamine, while the phosphorylated eIF2α (p-eIF2α) was not altered **(E,F)**. Band intensity of p-mTOR, mTOR, p-4EBP1, total 4EBP1, BiP and p-eIF2α was normalized by the respective GAPDH band and mean of three independent experiments was plotted with respective CI95 **(B, D, and F)**. ^*^no overlap of CI95 between indicated group and control group (0 μM).

The treatment with 50 and 100 μM of dopamine did not alter the total amount of PrP^C^ (Figures 2A,B). However, the fast-migrating band (#) was decreased with 100 μM of dopamine (Figures 2A,C). This band probably represents newly synthesized unglycosylated form rather than N-terminally truncated PrP^C^ because this band migrated close to 26 kDa marker and was recognized by SAF32, an antibody that binds to octa-repeat region located in N-terminal PrP^C^. The specific reduction of this immature form can indicate that dopamine toxicity might have affected protein synthesis. Thus, we verified the level of mTOR (a master regulator of growth and protein synthesis) together with 4EBP1 (a repressor of cap-dependent translation upon dephosphorylation). As shown in Figures 3A–D, phosphorylation of mTOR and 4EBP1 was significantly decreased by 100 μM of dopamine. Also, faster migration of total 4EBP1 bands indicated lower degree of phosphorylation. These data indicate that protein synthesis might be compromised in this condition. In addition, the expression level of ER chaperone BiP was increased with 50 μM of dopamine (Figures 3E,F), suggesting augment of misfolded proteins in ER. However, at 100 μM, BiP expression returned to control level (Figures 3E,F), presumably due to the attenuated protein synthesis. On the other hand, eIF2-α phosphorylation was not altered by dopamine treatment (Figures 3E,F).

**Figure 4:**
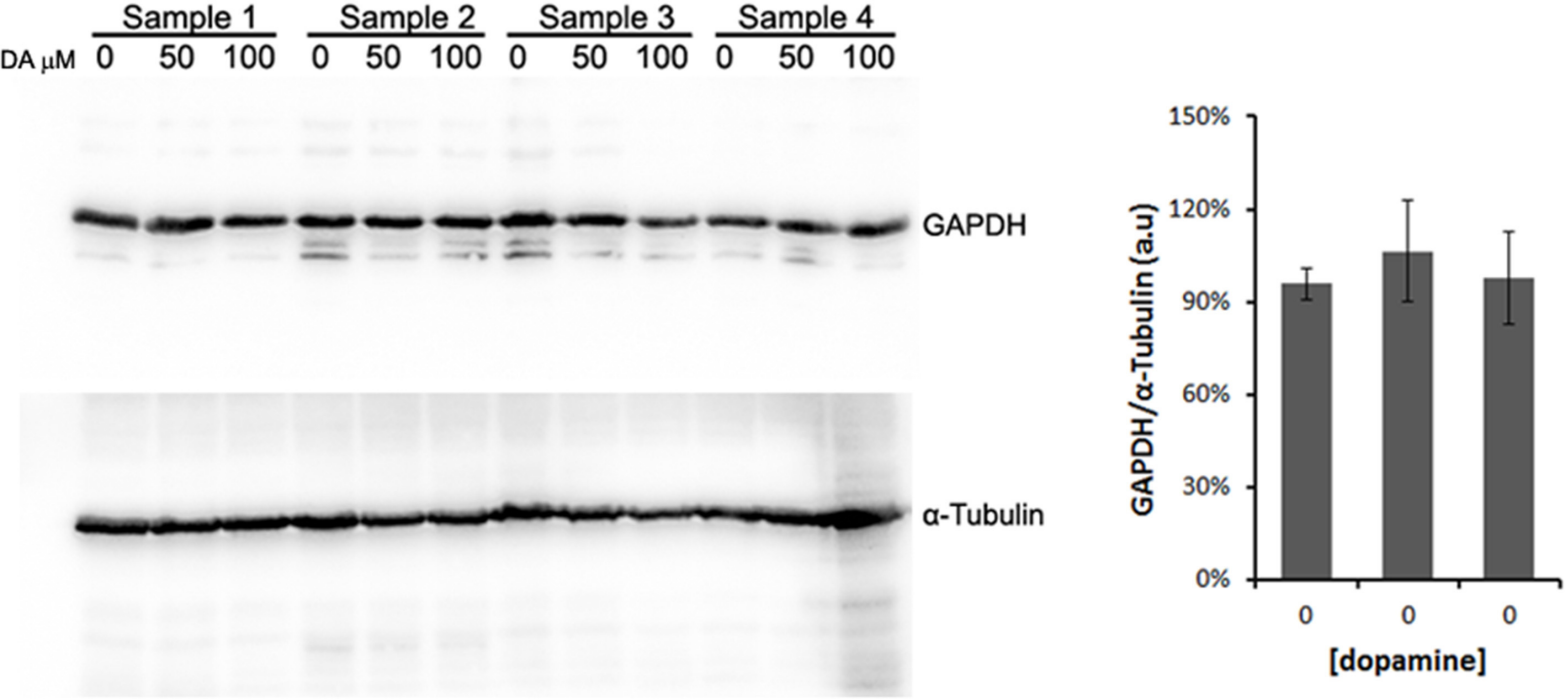
**Validation of GAPDH as loading control**. N2a cells were treated with dopamine at designated concentrations and cell lysates were used to evaluate the expression levels of GAPDH and α-tubulin in four independent samples. The band intensity of GAPDH was normalized by the intensity of α-tubulin. Mean normalized intensity was plotted with respective CI95.

All protein fold changes were calculated based on the loading control GAPDH. Thus, to ensure the constitutive expression of GAPDH in our experimental conditions, we compared the GAPDH expression to α-tubulin, another frequently used loading control. As shown in Figure 4, GAPDH expression was not significantly changed by dopamine treatment.

Altogether, these data indicate that 100 μM of dopamine compromises cellular viability, redox balance and protein synthesis, which may affect the synthesis of PrP^C^.

### Dopamine reduced the solubility of PrP^C^ and induced its accumulation in autophagosomes

**Figure 5:**
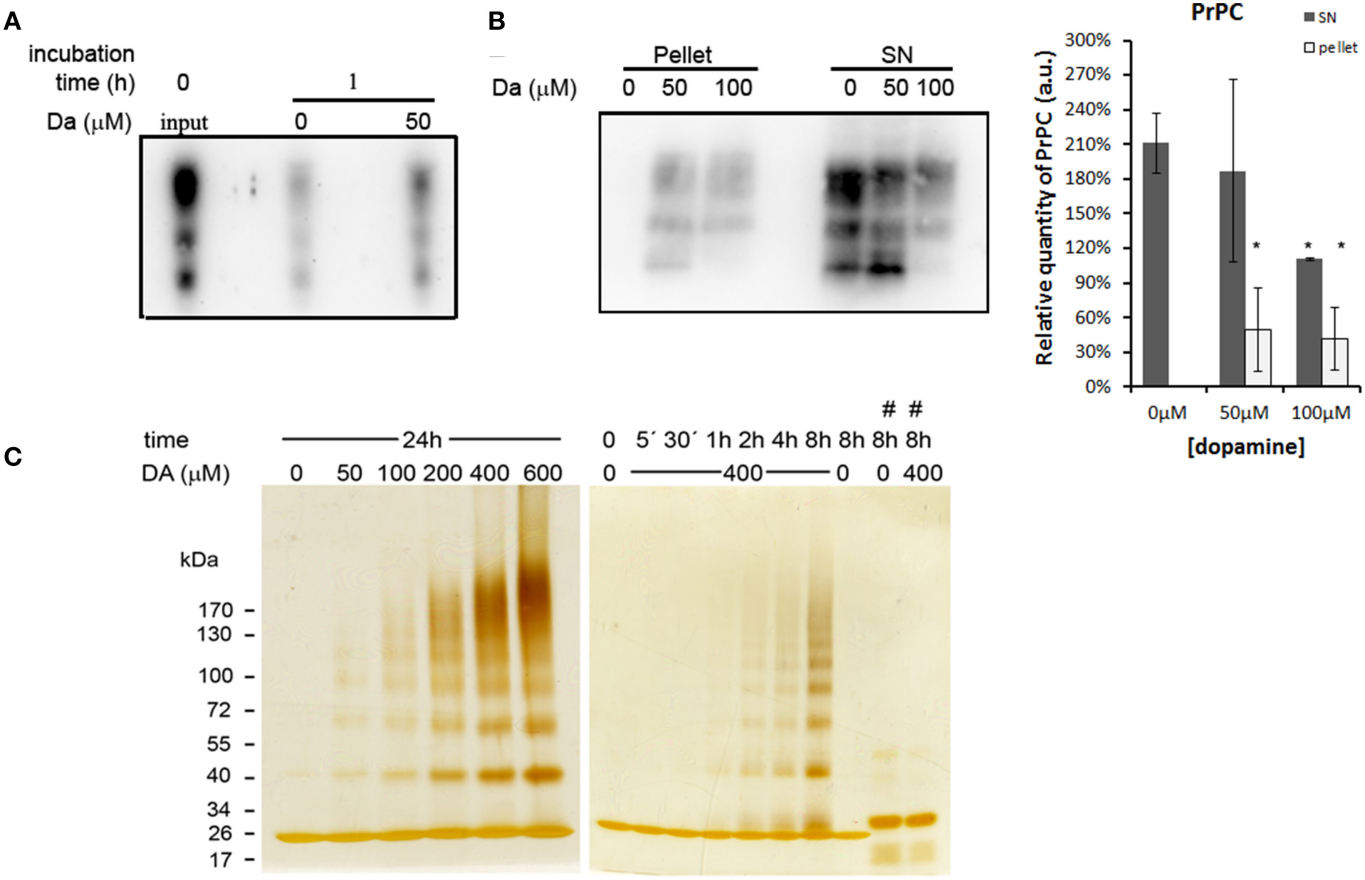
**Dopamine reduced turnover and solubility of PrP^C^**. Cell surface proteins was biotinylated and chased at time zero (input) or after 1 h incubation in the presence or absence of 50 μM of dopamine. After 1 h, dopamine treated cells showed higher amount of remaining PrP^C^ compared to untreated cells **(A)**. After dopamine treatment, insoluble proteins in sarkosyl 1% were recovered by ultracentrifugation. Higher amounts of PrP^C^ were recovered in pellet fractions and lower amount in supernatants (SN), indicating lower solubility **(B)**. Mean band intensity of three independent experiments was plotted with respective CI95. Dark gray bars represent SN and light gray bars represent pellet quantification **(B)**. ^*^no overlap of CI95 between indicated group and control group (0 μM). rPrP (10 μg) was incubated with designated concentration of dopamine (DA) for 24 h or with 400 μM of dopamine for indicated times **(C)**. When 400 μM of metabisulfite (#) was added, the oligomerization was prevented **(C)**. rPrP monomer migrated close to 26 kDa marker. Three independent experiments were performed and representative images are shown.

An intriguing fact is that even with reduced protein synthesis, we did not observe a significant reduction of the mature form of PrP^C^. One possibility is that dopamine treatment may reduce the turnover of PrP^C^. To test this hypothesis, we biotinylated cell surface protein and observed a turnover of PrP^C^. After 1 h of incubation, a substantial amount of biotinylated PrP^C^ was degraded in both dopamine-treated (50 μM) and untreated cells compared to input (Figure 5A, comparing second and third lane with first lane). However, a higher amount of biotinylated PrP^C^ remained in dopamine-treated cells compared to untreated cells, indicating that dopamine treatment reduced PrP^C^ turnover (Figure 5A, comparing second lane with third lane). To verify whether this reduced turnover is due to the altered biochemical characteristics, we evaluated the solubility of PrP^C^ in 1% sarkosyl. As shown in Figure 5B, an increased amount of PrP^C^ was recovered in insoluble pellet fraction after treatment with 50 and 100 μM of dopamine (Figure 5B). To further evaluate the effects of dopamine on PrP^C^ aggregation, *in vitro* experiments were conducted using recombinant mouse PrP^C^ (rPrP). Dopamine induced the formation of SDS-resistant higher-order oligomers of rPrP in a time and concentration-dependent manner (Figure 5C). Oligomerization was prevented in the presence of antioxidant (sodium metabisulfite 400 μM) (Figure 5C, last two lanes). These results indicate that oxidative metabolites of dopamine can induce the accumulation of insoluble PrP^C^. In the presence of metabisulfite, monomer rPrP migrated slowly. Even after reduction of proteins with DTT, sulfhydryl groups can be readily reoxidized and form disulfide bond (Wall, 1971), and reduced recombinant PrP migrates more slowly than oxidized form (Lee and Eisenberg, 2003). Likely, metabisulfite prevented the reoxidation of sulfydryl groups, resulting in slower migration (Figure 5C).

**Figure 6:**
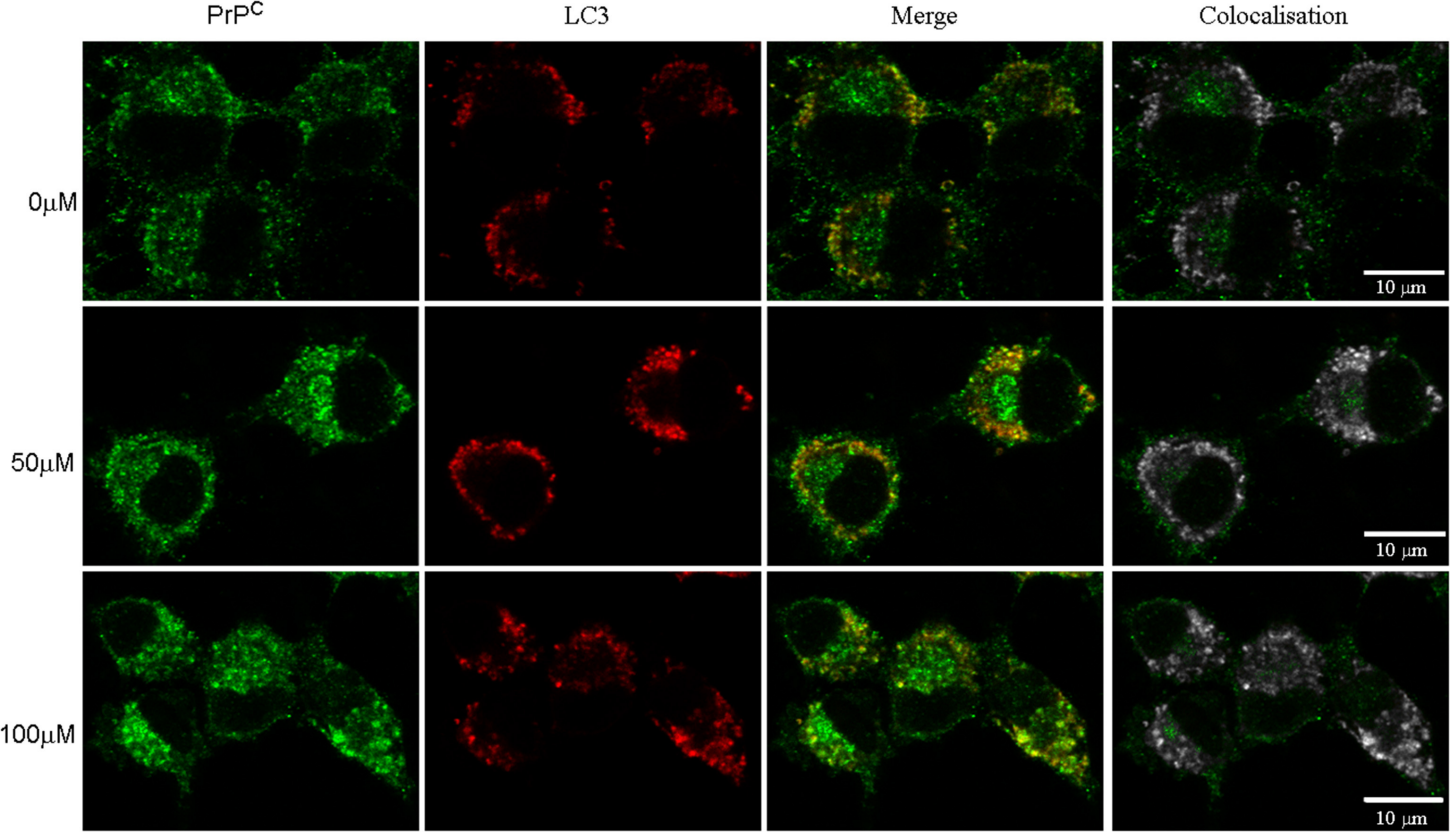
**Dopamine induced PrP^C^ sorting to autophagosomes**. After dopamine treatment, endogenous PrP^C^ (green) and LC3-I/LC3-II (red) was detected using specific antibodies. Third column shows merged images. Right column shows colocalized pixels in gray scale. Scale bar = 10 μm.

**Figure 7:**
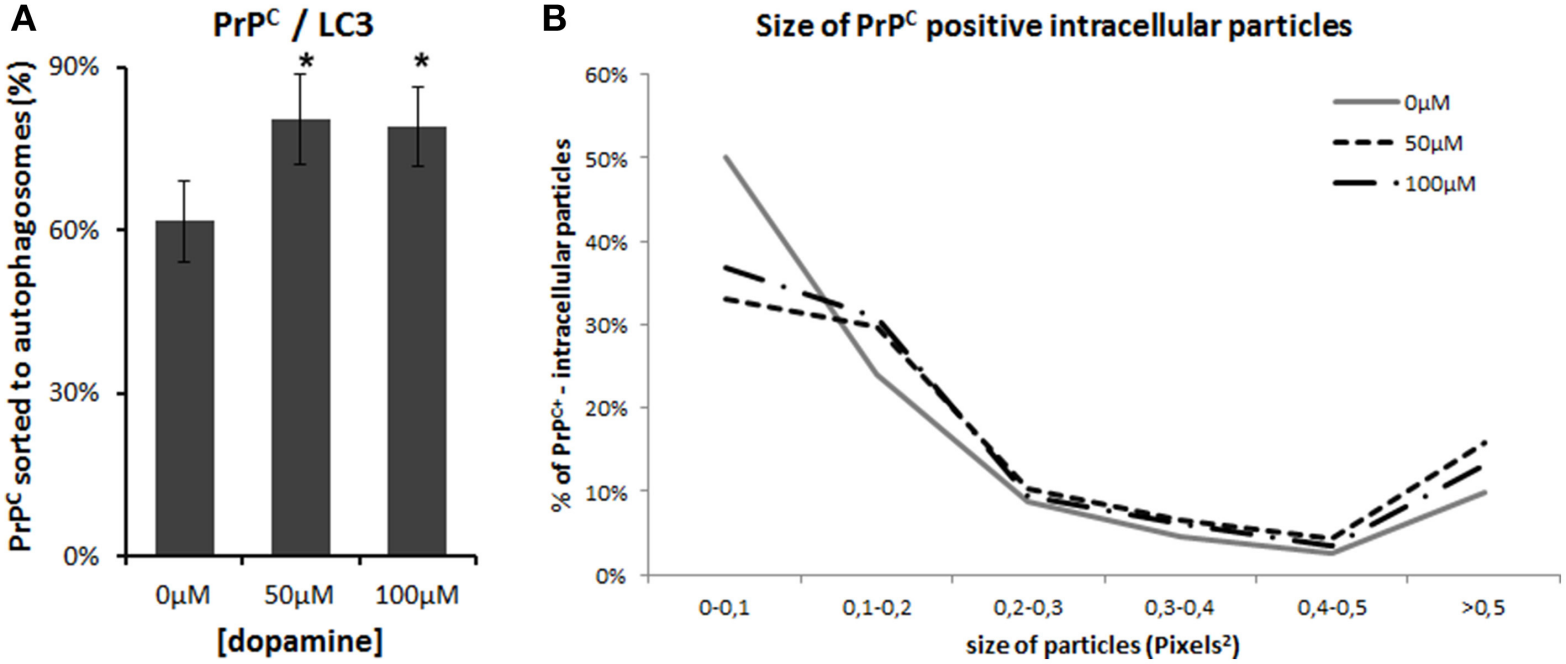
**Image analysis**. Percentage of PrP^C^-positive area that colocaize with LC3-II was calculated using colocalization threshold plugins of Image J. The graphs represent mean of 5 to 6 images with respective CI95 **(A)**. ^*^ no overlap of CI95 between indicated group and control group. The area of PrP^C^-positive intracellular vesicles was measured in pixel^2^ using Image J and was categorized by their area. Percentage of each category was calculated. **(B)**. Dopamine treated cells (dotted lines) showed lower percentage of smaller vesicles and higher percentage of vesicles that measured more than 0.5 pixel^2^.

Autophagy is one of the pathways that degrade prion protein (Homma et al., 2014). Since dopamine treatment induced accumulation of insoluble PrP^C^, we evaluated whether these aggregates were sorted to autophagosomes, labeled by LC3-II (an autophagosome membrane associated protein). As shown in Figure 6, the degree of colocalization between PrP^C^ and LC3-II was increased by dopamine treatment (Figures 6, 7A). Of note, the intracellular organelles that contained PrP^C^ were frequently enlarged in cells treated with dopamine (Figures 6, 7B) and they were mostly positive for LC3-II staining (Figure 6).

**Figure 8:**
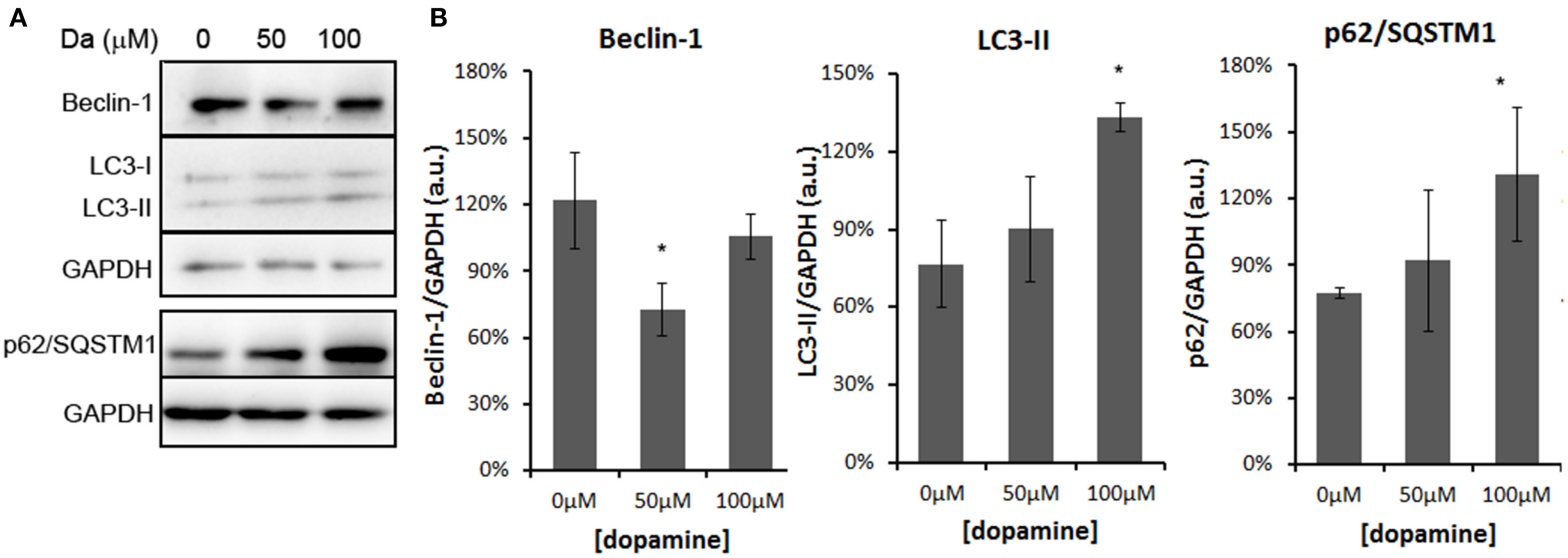
**Dopamine treatment induced accumulation of autophagosomes**. The level of Beclin-1 was reduced by 50 μM of dopamine and restored to control level at 100 μM, while p62/SQSTM1 and LC3-II was increased by 100 μM of dopamine **(A)**. Beclin-1 and LC3-II were analyzed in the same gradient gel. Band intensity of Beclin-1, p62/SQSTM1, and LC3-II was normalized by the respective GAPDH band and mean of three independent experiments was plotted with respective CI95 **(B)**. ^*^no overlap of CI95 between indicated group and control group (0 μM).

In addition, treatment of the N2a cells with 100 μM of dopamine increased the levels of p62/SQSTM1 and LC3-II (Figures 8A,B). The former transports ubiquitinated proteins to the autophagosome and remains associated to LC3-II, which is produced by lipidation of LC3-I (Ravikumar et al., 2010). The accumulation of both proteins occurred without the induction of Beclin-1, a component of the complex that initiates the formation of autophagosome (Figures 8A,B) (Ravikumar et al., 2010). Thus, these data suggest that the accumulation of autophagosomes was not due to the activation of autophagy, but rather the failure in further processing of autophagosomal cargo. The immunofluorescence data also showed the accumulation and enlargement of LC3-II positive-vesicles (Figure 6).

Overall, these results indicate that toxic concentration of dopamine altered the solubility of PrP^C^ and promoted its accumulation in autophagosomes, affecting autophagic flux and attenuating protein synthesis.

## Discussion

Dopamine is an important neurotransmitter that controls several functions such as physical movement, emotional process and alertness (Kauer and Malenka, 2007). In spite of its importance in physiology, dopamine metabolism has been pointed out as the main etiology of Parkinson's disease due to the inherent instability and reactivity of dopamine and its metabolites, capable to induce oxidative damage in biomolecules (Hastings, 2009). In this study, we demonstrated that dopamine treatment altered the solubility of PrP^C^ and promoted its accumulation in autophagosomes in neuronal cells. Auto-oxidation of dopamine also induced the formation of SDS-resistant oligomers of unglycosylated recombinant prion protein. Previous studies have demonstrated that inhibition of complex glycosylation during synthetic pathway or expression of unglycosylated PrP facilitated its conversion into PrP^Sc^ (Korth et al., 2000; Winklhofer et al., 2003). In our study, dopamine induced aggregation of both glycosylated neuronal PrP^C^ and unglycosylated rPrP, but with different biochemical characteristics regarding to the resistance to SDS.

Although the conversion of PrP^C^ into abnormal prion protein (PrP^Sc^) is an essential event for the development of TSE, molecular mechanisms of conformational transition between PrP^C^ and PrP^Sc^ are poorly understood. Nevertheless, previous studies have demonstrated that PrP^Sc^-like aggregates were found in normal brains at low levels (Yuan et al., 2006). Glycosylation pattern, retrograde transport from ER to cytosol and inhibition of proteosomal activity might contribute to this spontaneous production of PrP^Sc^-like aggregates (Korth et al., 2000; Yedidia et al., 2001; Ma and Lindquist, 2002; Winklhofer et al., 2003). The aggregation of PrP^C^ in response to redox alteration has also been proposed as a cytoprotective mechanism (Das et al., 2010). However, if the aggregates are not effectively degraded by autophagy, other proteins might co-aggregate causing cytotoxicity (Das et al., 2010). In this study, we observed that dopamine treatment increased the amount of LC3-II and p62/SQSTM1, both involved in autophagosomal cargo recruitment. Concomitantly, PrP^C^ was also accumulated in autophagosomes. These data indicate that the degradation of autophagosomal cargo by lysosome was not effective. Therefore, it is plausible to speculate that the reduced cell viability observed after dopamine treatment is at least partially due to the accumulation of PrP^C^ aggregates in autophagosomes. Similarly, PrP^C^ aggregation might have negatively affected protein synthesis since a previous study has demonstrated that protein synthesis was corrupted in N2a cells infected with prions (Roffe et al., 2010).

Endocytosis of PrP^C^ can be stimulated by several compounds such as Cu^2+^, nucleic acids and heme (Lee et al., 2001, 2007; Kocisko et al., 2006). An intriguing fact is that these compounds can alter the structure of PrP^C^ and confer cytotoxicity at high concentration (Thakur et al., 2011; Macedo et al., 2012). These findings suggest that PrP^C^ might function as a scavenging receptor for toxic molecules. Dopamine can induce toxicity by rapid auto-oxidation producing reactive dopamine-quinone (Asanuma et al., 2003; Chen et al., 2008). Binding of toxic molecules to PrP^C^, including dopamine metabolites, might induce structural alterations, which in turn, may trigger endocytosis for further degradation. Frequent binding of toxic molecules that cause conformational alterations of PrP^C^ can eventually overload the degradation pathways, instigating the accumulation of protein aggregates.

Certain conditions, such as stress, drug addiction, iron deficiency, L-DOPA treatment or schizophrenia are known to increase dopamine metabolism (Nelson et al., 1997; de la Fuente-Fernandez et al., 2004; Elliott and Beveridge, 2005; Kim et al., 2005). Our findings raise a possibility that chronic exposure to these conditions may facilitate PrP^C^ aggregation. Possibly, dopamine and its oxidative metabolites can have more general roles in other protein aggregation, but the specificity of the target can be ruled by subcellular compartmentalization, brain region specificity and susceptibility of aggregation. Identification of endogenous metabolites that induce aggregation of a specific protein can contribute to better understanding of idiopathic neurodegenerative diseases and provide new molecular targets for treatment.

## Author contributions

Marcio H. M. da Luz conducted all experiments except rPrP oligomerization and analyzed the results. Italo T. Peres performed rPrP oligomerization assay. Tiago G. Santos acquired confocal images. Vilma R. Martins participated in study design and interpretation of data and revised the manuscript. Marcelo Y. Icimoto purified rPrP. Kil S. Lee participated in study design, data analysis and interpretation, and wrote the manuscript.

### Conflict of interest statement

The authors declare that the research was conducted in the absence of any commercial or financial relationships that could be construed as a potential conflict of interest.

## Abbreviations

ROS: Reactive oxygen species
PrP^C^: Cellular prion protein
PrP^Sc^: abnormal prion protein
SDS: Sodium dodecyl sulfate
mTOR: Mammalian target of rapamycin
4EBP1: 4E binding protein 1
ER: Endoplasmic reticulum
eIF2-α: Eukariotic initiation factor 2-α
p62/SQSTM1: Sequestosome 1
LC3: Microtubule-associated protein 1 light chain 3
TSE: Transmissible spongiform encephalopathies.
